# Correction: Ribosome profiling reveals pervasive and regulated stop codon readthrough in *Drosophila melanogaster*

**DOI:** 10.7554/eLife.03178

**Published:** 2014-05-06

**Authors:** Joshua G Dunn, Catherine K Foo, Nicolette G Belletier, Elizabeth R Gavis, Jonathan S Weissman

Dunn JG, Foo CK, Belletier NG, Gavis ER, Weissman JS. 2013. Ribosome profiling reveals pervasive and regulated stop codon readthrough in *Drosophila melanogaster*. *eLife* **2**:e01179. doi: 10.7554/eLife.01179. Published 3 December 2013

In the published article, in the section ‘Lysate preparation’ within the Materials and methods, the sentence ‘To start the experiment, cells were treated for 20 min with 0.01 vol 2 mg/ml emetine…’ should have read ‘To start the experiment, cells were treated for 2 min with 0.01 vol 2 mg/ml emetine…’.

This has been corrected.

